# Research progress on occupational therapy for disorders of consciousness in the context of neurorehabilitation: visual analysis based on CiteSpace

**DOI:** 10.3389/fneur.2026.1795682

**Published:** 2026-05-07

**Authors:** Jinqin Zhang, Xinyue Yan, Fubiao Huang, Ying Dong, Yudi Huang, Ying Zhang

**Affiliations:** 1 Department of Occupational Therapy, China Rehabilitation Research Center, Beijing, China; 2 Faculty of Rehabilitation, Capital Medical University, Beijing, China; 3 Otawara Campus, International University of Health and Welfare, Otawara, Japan; 4 Faculty of Rehabilitation, Shandong University of Traditional Chinese Medicine, Jinan, China; 5 Faculty of Rehabilitation Medicine, Wenzhou Medical University, Wenzhou, China; 6 Department of Rehabilitation Information Research, China Rehabilitation Science Institute, Beijing, China; 7 WHO-FIC Collaborating Center, Beijing, China; 8 Faculty of Rehabilitation, Shandong University of Traditional Chinese Medicine, Jinan, Shandong, China; 9 School of Rehabilitation Medicine, Shandong Second Medical University, Weifang, Shandong, China

**Keywords:** CiteSpace, disorders of consciousness, occupational therapy, review, bibliometric analysis

## Abstract

**Objective:**

This study aimed to analyze recent research and emerging trends in occupational therapy for disorders of consciousness.

**Methods:**

We employed bibliometric methods to retrieve relevant English literature on occupational therapy for disorders of consciousness from Web of Science Core Collection (WOSCC) and China National Knowledge Infrastructure (CNKI), including publications up to July 10, 2025. CiteSpace 6.2. R1 was utilized to generate knowledge maps, focusing on authors, institutions, countries, and keywords.

**Results:**

We identified 526 articles in English. Among contributing authors, Laureys, Steven demonstrated the highest productivity (15 publications). The United States and Institut National de la Sante et de la Recherche Medicale were the leading contributing countries and institutions. “Archives Of Physical Medicine And Rehabilitation” was the most influential journal with a total of 300 citations. High-frequency keywords include “vegetative state” and“disorders of consciousness.”

**Conclusion:**

This 10-year bibliometric analysis of occupational therapy for disorders of consciousness research identifies priority areas for future studies.

## 1 Introduction

Disorders of consciousness (DOCs) encompass pathological alterations in states of consciousness resulting from structural or functional damage to the neural networks governing arousal and awareness. These disorders include coma, the vegetative state (VS, also known as unresponsive wakefulness syndrome (UWS)), and the minimally conscious state (MCS), all of which are defined by standardized diagnostic criteria (1–5). A recent addition to the diagnostic classification scheme of patients with DOC is the concept of cognitive motor dissociation (CMD) (6), also known as covert consciousness. Previous research has indicated that the incidence of VS/UWS is 525 cases per million population (PMP) in various countries. Previous research has also suggested that the prevalence of MCSs is tenfold greater than that of VS (7). DOC significantly affects patients’ survival rates, ability to perform activities of daily living (ADL), quality of life, employment, and relationships postinjury, resulting in considerable caregiving demands and substantial societal medical resource expenditures. Treatment goals may vary over the course of recovery, with the end goal being improving the occupational performance, participation, and quality of life of the person with DOC. While pharmacological agents and neuromodulatory interventions remain the cornerstone treatments (8, 9), noninvasive approaches such as occupational therapy have become increasingly important.

Occupational therapy intervention approaches may shift throughout the recovery process, depending on the level of severity and recovery. Occupational therapy interventions may include establishing or restoring a skill or ability, compensating, adapting the context or activity, maintaining function with support to preserve abilities, and preventing occupational performance limitations (10). According to the American Occupational Therapy Association, occupational therapy practitioners are uniquely qualified to “assess the client’s ability to engage in occupational performance” and provide interventions to address those limitations (11). Standard occupational therapy interventions to address limitations in occupational performance include the use of occupation-based goals, client-centered practices, interventions to support occupation, education, training, advocacy, group interventions, virtual service delivery, and therapeutic use of occupations and activities (11, 12). Many studies have investigated the application of occupational therapy in the rehabilitation of DOC. However, existing studies focus on the current status, influencing factors, or intervention validation, while systematic analyses of knowledge architecture, disciplinary evolution, and international collaboration patterns remain lacking.

Bibliometrics quantifies the development direction of specific topics and discovers the knowledge relationships between research fields by screening and analyzing large amounts of data and applying visualization techniques, thereby predicting the development trends and potential directions of future research (13). As an interactive visualization tool that integrates information visualization, bibliometrics, and data mining algorithms, CiteSpace intuitively presents the internal relationships among data in the form of maps, reveals the knowledge structure of the target research field, and highlights current research trends (14, 15). In this study, CiteSpace is used for visualization analysis to explore the intellectual foundations, emerging hotspots, and frontier trends of occupational therapy in the field of DOC rehabilitation from various dimensions. These findings may guide evidence-based rehabilitation practices, policy design, interdisciplinary resource integration, and future research prioritization in stroke rehabilitation.

## 2 Materials and methods

### 2.1 Data source

This descriptive bibliometric study investigates research trends, collaborative models, and thematic evolution in occupational therapy applications for consciousness disorder rehabilitation. The search results from the WOSCC and CNKI were exported in plain text format with the fields “Full Record and Cited References” selected (16). Consequently, the findings possess authoritative, appropriate, and representative validity.

### 2.2 Search strategy

The literature search was performed on July 10, 2025. The search strategy focused on studies related to the theme “Application of Occupational Therapy in the Rehabilitation of Consciousness Disorders” with only articles and reviews included. The search covered publications indexed in Web of Science from January 2015 to July 2025. The detailed search strings are provided in Supplementary Table 1.

### 2.3 Selection criteria

Inclusion criteria were as follows: (1) articles focused on DOC and OT, (2) publication between January 2015 and July 2025, (3) document type: original research articles or reviews, (4) research design: clinical trial, review, systematic review and meta-analysis, and (5) language: English or Chinese.

This review adopts the following operational definition for the topic of “OT”: (1) The research content encompasses theoretical frameworks, assessment tools, intervention programs, or outcome measures in OT. (2) Although studies may utilize non-occupational therapy-specific methods such as neuroimaging (fMRI), electrophysiology (EEG), diagnostic assessments, or prognostic indicators, their research objectives aim to enhance individual participation, functional performance, and quality of life, which aligns with the core goals of OT. (3) Literature that meets any of the above criteria and focuses on functional improvement in rehabilitation populations is defined as occupational therapy-related research.

Exclusion criteria were as follows: (1) the research topic was not related to DOC or OT; (2) incomplete or duplicated data; (3) early access publications; (4) Retracted publications; and (5) conference proceedings, technical achievements, books, patents, newspapers, letters, editorials, etc.

### 2.4 Data screening and extraction

Data collection was conducted by two researchers experienced in systematic reviews and meta-analyses. Two independent reviewers screened titles and abstracts to assess eligibility. Data filtering and extraction. Preliminary document screening was executed through title or abstract assessment within the Web of Science platform and later exported in plain textual format containing the complete Web of Science record and cited references. Each record contained author credential, publication title, periodical source, abstract, keywords, references, institution, funding, and citation information. The exported text was named “download.txt” to ensure compatibility with CiteSpace 6.4. R1&R2, where the data were imported, cleaned, and deduplicated, followed by format conversion (17).

### 2.5 Data analysis

Software tools: CiteSpace 6.2. R1and Microsoft Excel.

Analysis parameters: Time slicing = 1 year, Timeframe = 2015–2025.

Network types:

Collaboration networks (countries, institutions, and authors).
Co-citation networks (journals, references, and authors).
Keyword cooccurrence, clustering, burst analysis and timeline analysis.

Methodological details:

Microsoft Excel-generated annual cumulative publication counts.
CiteSpace 6.2. R1extracted three network types—collaboration, co-citation, and co-occurrence. In visualizations, the following is true:
Node types include authors, institutions, countries, keywords, cited references, cited authors, and cited journals.
Node selection criteria utilize the default Top N value and apply pruning via the Pathfinder and Pruning Sliced Networks algorithms, while all other options remain at their defaults.
Node size corresponds to frequency.
Lines reflect cooperative, co-occurrence, or co-citation relationships between nodes.
Line thickness reflects co-occurrence strength.
Nodes with a centrality score greater than 0.1 are marked with a purple ring, indicating their pivotal role in connecting research subfields (18–20).

### 2.6 Ethical considerations

As this study involved secondary analysis of bibliometric data from publicly available databases, no ethical approval was needed. All procedures complied with Web of Science usage policies and academic integrity standards.

## 3 Results

**Figure 1:**
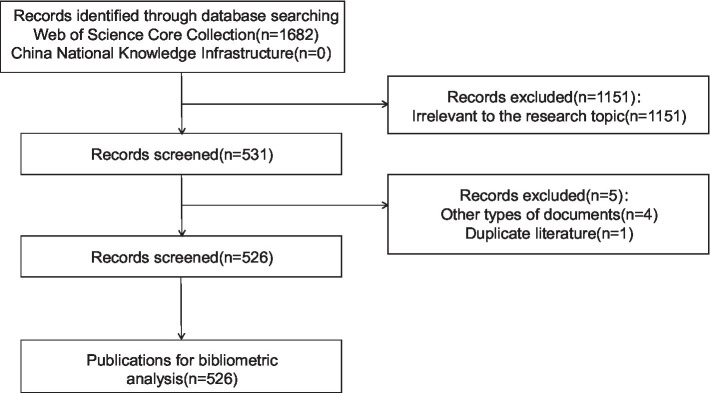
Flow diagram of study selection.

The literature screening workflow is shown in Figure 1. The initial search yielded 1,682 publications, with 526 meeting the inclusion criteria.

### 3.1 Annual publication analysis

**Figure 2:**
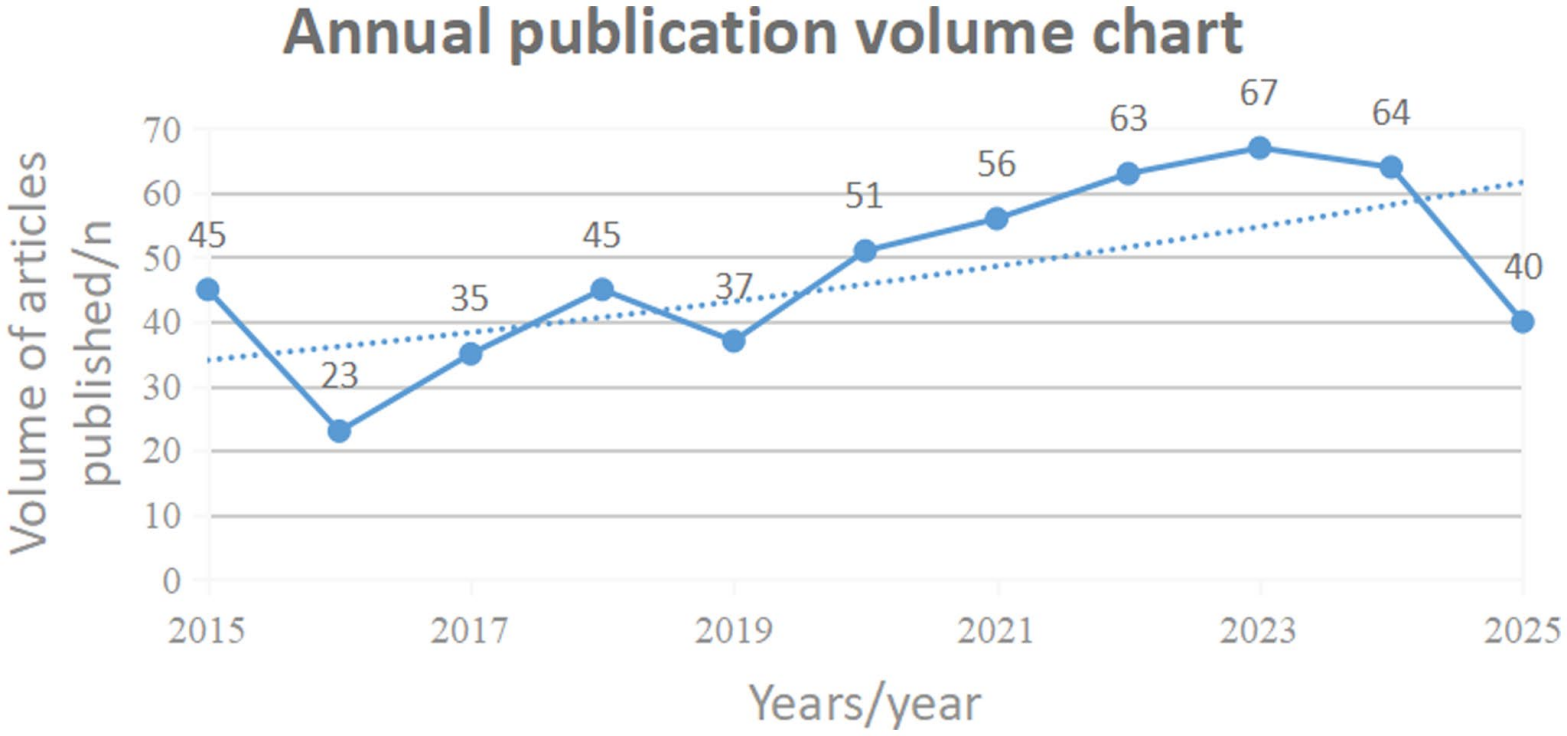
Annual publication volumn chart.

A total of 1,682 articles were initially retrieved, including 1,682 from the WOSCC and 0 from the CNKI. Finally, 526 articles met the inclusion criteria. The number of publications in each period directly reflects the development trend of scientific knowledge in a specific field. The annual publication trends of research on the application of occupational therapy in consciousness disorders from January 2015 to July 2025 are shown in Figure 2. The average annual publication volume of related literature was 52.6 articles. Although there were minor declines in 2015–2016, 2018–2019, and 2023–2025, the overall trend over the past decade showed a fluctuating upward trajectory, reaching its peak in 2023 (*n* = 67).

### 3.2 Analysis of countries

**Figure 3:**
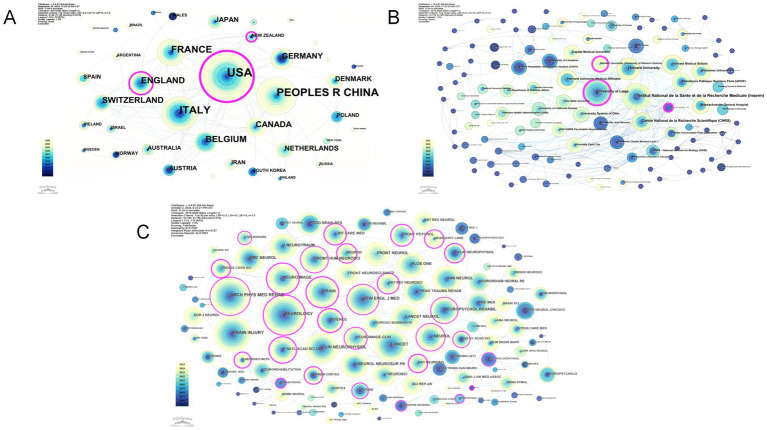
Bibliometric landscape of research. **(A)** Country/region collaboration network. **(B)** Institutional collaboration network. **(C)** The co-occurrence map of cited journals.

This study employs CiteSpace to analyze co-occurrence relationships among countries/regions, aiming to reveal collaboration patterns and development trends across the research field through a visualized cooperation network. Using “country” as nodes with a Top *N* = 10 threshold, the generated network comprises 40 nodes, 126 connections, and a network density of 0.1615 (Figure 3A).

In terms of the number of published papers, the top 10 countries/regions are the United States, the People’s Republic of China, Italy, France, England, Belgium, Canada, Switzerland, Germany, and Denmark (Table 1), indicating that these countries/regions lead in terms of paper output. The United States maintains its position as the network core with an absolute advantage in publication volume and an intermediary centrality of 0.87, demonstrating its absolute influence in this field. Apart from the United States, other countries have not published more than 100 papers, reflecting that there is still room for improvement in knowledge output. In terms of citation half-life, the top 10 countries/regions are China, Denmark, Argentina, Brazil, the United States, Canada, France, Russia, New Zealand, and England (Table 1). The citation half-life values of these countries are greater than 4, indicating that their academic achievements in relevant research fields have a long-lasting impact and that their related literature continues to be frequently cited and receives sustained attention from the academic community. In terms of centrality, the top 10 countries/regions are the United States, New Zealand, England, Italy, Belgium, Australia, the Netherlands, Poland, Canada, and Norway (Table 1). Among them, the United States, England, Italy, and Belgium not only have high publication volumes but also high centrality values, suggesting that their scientific achievements and collaborative relationships strongly influence knowledge dissemination and collaboration within the field. Although New Zealand has a relatively low number of publications, its intermediary centrality value exceeds 0.1, ranking second. This ranking indicates that New Zealand plays a pivotal role in this research field, with its network influence far surpassing its publication volume.

**Table 1:** Top 10 countries/regions by publication output, citation half-life and centrality.

Rank	Sort by no. of articles published	Frequency	Sort by citation half-life	Citation half-life	Sort by centrality	Centrality
1	USA	148	Peoples R China	6.5	USA	0.87
2	Peoples R China	94	Denmark	6.5	New Zealand	0.17
3	Italy	80	Argentina	6.5	England	0.13
4	France	53	Brazil	6.5	Italy	0.07
5	England	37	USA	5.5	Belgium	0.06
6	Belgium	35	Canada	5.5	Australia	0.05
7	Canada	31	France	5.5	Netherlands	0.05
8	Switzerland	28	Russia	5.5	Poland	0.05
9	Germany	25	New Zealand	4.5	Canada	0.04
10	Denmark	17	England	4.5	Norway	0.04

From a geographical perspective, the collaboration network exhibits distinct regional clustering. The United States maintains close ties with European countries such as England, Italy, and France, while among Asian nations, only China stands out in terms of citation half-life, with no other Asian countries ranking high in other aspects. Moreover, the influence of China’s scientific achievements and collaborative relationships on knowledge dissemination and collaboration within the field remains insufficient, indicating an imbalance in the geographical distribution of this research domain. Future cross-regional cooperation, particularly with Asian countries, holds immense potential for development.

### 3.3 Institutional analysis

By employing CiteSpace for institutional co-occurrence analysis, we can visualize the collaborative network among research institutions, effectively revealing the cooperation patterns within a specific field. This approach provides direct evidence for identifying core research forces and optimizing the allocation of research resources. When “institutions” are used as nodes and a threshold of Top *N* = 10 is selected, the resulting institutional network map comprises 119 nodes, 347 connections, and a network density of 0.0494 (Figure 3B). The top 10 institutions in terms of publication volume are listed in Table 2. These results indicates that although the publication volume of University of Liège is not the highest, it plays a crucial intermediary and bridging role in the collaborative network. Among the top 10 institutions by centrality, both the University of Liège (0.17; Belgium) and Western University (0.15; Canada) have centrality values exceeding 0.1. These institutions engage in cross-institutional collaboration through fewer publications, serving as irreplaceable bridges. Therefore, researchers should not only ensure their own publication output but also strengthen interinstitutional cooperation, leveraging cross-regional and cross-institutional collaboration to further integrate resources and enhance research output and impact.

**Table 2:** Top 10 institutions by publication output and centrality.

Rank	Sort by no. of articles published	Frequency	Sort by centrality	Centrality
1	Institut National de la Sante et de la Recherche Medicale	41	University of Liege	0.17
2	Centre National de la Recherche Scientifique	27	Western University	0.15
3	University of Liege	25	Assistance Publique Hopitaux Paris	0.09
4	Harvard University	23	Columbia University	0.08
5	Harvard Medical School	20	Institut National de la Sante et de la Recherche Medicale	0.07
6	Harvard University Medical Affiliates	20	Massachusetts General Hospital	0.07
7	Assistance Publique Hopitaux Paris	19	Centre National de la Recherche Scientifique	0.06
8	Sorbonne Universite	19	Hopital Universitaire Pitie-Salpetriere - APHP	0.06
9	Massachusetts General Hospital	15	Harvard Medical School	0.05
10	Capital Medical University	15	Capital Medical University	0.04

### 3.4 Analysis of cited journals

Using CiteSpace for co-occurrence analysis of cited journals can reveal the most important and core knowledge sources in this field, thereby providing key references for researchers to submit papers and track cutting-edge developments. With “cited journals” as nodes and a threshold of Top N = 50, the resulting network map of cited journals includes 120 nodes, 136 connections, and a network density of 0.019 (Figure 3C). The top 10 journals by citation frequency are listed in Table 3. These findings demonstrate that research in this field is grounded in rigorous clinical evidence chains (diagnosis–assessment–intervention) and supported by advanced assessment technologies, focusing on mechanism exploration and clinical validation. The five journals with the greatest centrality were Neuroimage (IF = 4.5; centrality, 1.04), Proceedings of the National Academy of Sciences (IF = 9.1; centrality, 0.98), Journal of Neuroscience Methods (IF = 2.7; centrality, 0.76), Science (IF = 45.8; centrality, 0.58), and Nature Neuroscience (IF = 21.2; centrality, 0.5) (Table 3). Among the top 10 journals, all exhibited an intermediary centrality greater than 0.01, indicating their prominent role as hubs in connecting different research clusters.

**Table 3:** Top 10 cited journals by citation frequency and centrality.

Rank	Sort by no. of cited articles	Frequency	Sort by centrality	Centrality
1	Archives Of Physical Medicine And Rehabilitation	300	Neuroimage	1.04
2	Neurology	298	Proceedings of the National Academy of Sciences	0.98
3	Brain Injury	270	Journal of Neuroscience Methods	0.76
4	Lancet	227	Science	0.58
5	Clinical Neurophysiology	202	Nature Neuroscience	0.5
6	Brain	190	Brain	0.49
7	The New England Journal of Medicine	190	Trends in Cognitive Sciences	0.41
8	Neuroimage	180	Frontiers in Psychology	0.39
9	Public Library of Science ONE	159	Resuscitation	0.39
10	Annals of Neurology	159	Neurology	0.33

### 3.5 Analysis of authors and co-cited authors

CiteSpace co-occurrence analysis of authors can describe the core research forces and collaboration within a research field, which not only provides an intuitive representation of academic collaboration information but also offers data support for subsequent scientific research planning and team building. CiteSpace-based author citation analysis reveals the knowledge structure of a research field. When two authors are frequently cited by the same subsequent paper, they form a co-citation relationship, which typically indicates intrinsic connections in their research themes, methods, or theories. In cocitation networks, key authors in a field are often identified through such analysis. Authors who are frequently cited are generally considered to have greater influence than those with lower citation frequency.

**Figure 4:**
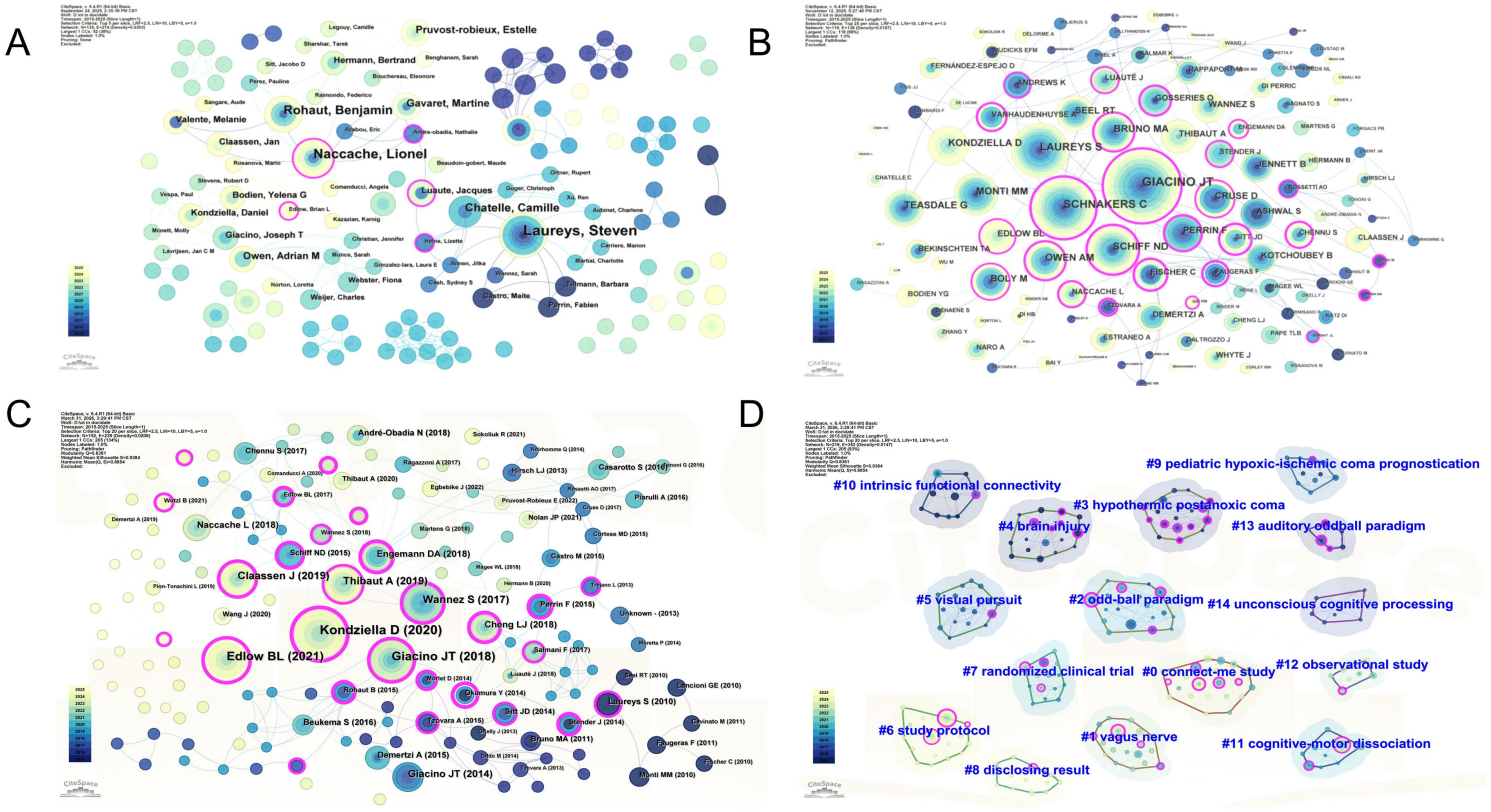
Bibliometric landscape of research. **(A)** Author collaboration network. **(B)** Co-citation network of cited authors. **(C)** The co-occurrence map of cited references. **(D)** The cluster map of cited reference (different colors represent different clusters).

When authors are used as nodes with a Top *N* = 5 threshold selection, the resulting author network graph comprises 135 nodes, 274 connections, and a network density of 0.0303 (Figure 4A). The collaborative network reveals distinct clusters marked by different colors, indicating limited cooperation among authors. This finding suggests the existence of multiple relatively independent yet closely connected small academic groups, while extensive cross-team and cross-institutional collaboration remains insufficient. Promoting communication between different groups will be key to future development. As shown in Table 4, the top 10 authors by publication volume are Laureys and Steven, who lead with 15 papers, followed by Naccache and Lionel, the two most prolific core authors in the field. Both Naccache and Lionel, as well as Luaute and Jacques, exhibit centrality values >0.1 (Table 4), highlighting their core contributions to the field.

According to Price’s law (M = 0.749 × √Nmax), a core author group can only form when core authors’ publications account for 50% of the total publications. By combining those authors with the greatest productivity, Laureys and Steven (Nmax = 15), the calculated M ≈ 2.9 indicates that core authors should be defined as those with ≥3 publications. There are 32 core authors who have collectively published 151 papers, accounting for 28.70% of the total number of publications in this field. This proportion is significantly lower than the 50% threshold proposed by Price’s law, suggesting that the core author group has not yet stabilized. This finding also reflects relatively independent research and limited collaboration among core authors.

When “cited authors” are used as nodes with a threshold of Top N = 25, the resulting citation network graph comprises 119 nodes, 138 connections, and a network density of 0.0197 (Figure 4B). The top 10 cited authors are listed in Table 5. The high-level citation frequency indicates that the research achievements of these 10 authors constitute the core knowledge base and methodological source for occupational therapy in the field of consciousness disorder rehabilitation. Further discussion of mediating centrality values can reveal authors who play a key “bridging” role in knowledge dissemination (Table 5). Although Sitt, Jacobo D. (centrality 1.13) and Faugeras, Frederic (centrality 0.92) do not have the highest total citation frequency, their mediating centrality ranks in the top two, indicating that their research serves as a critical link between different theoretical or methodological clusters, significantly promoting the interdisciplinary integration of neuroscience and clinical rehabilitation research. Notably, both Schnakers and Caroline (*n* = 162 citations; centrality, 0.74) and Schiff and Nicholas D. (*n* = 93 citations; centrality, 0.7) rank among the top five in terms of both citation frequency and mediating centrality. This finding highlights their dual role not only as prolific authorities in their fields but also as pivotal hubs bridging diverse research directions and advancing interdisciplinary knowledge integration.

**Table 4:** Top 10 authors by publication output and centrality.

Rank	Sort by no. of articles published	Frequency	Sort by centrality	Centrality
1	Laureys, Steven	15	Naccache, Lionel	0.16
2	Naccache, Lionel	12	Luaute, Jacques	0.13
3	Rohaut, Benjamin	9	Laureys, Steven	0.08
4	De lucia, Marzia	7	Bodien, Yelena G	0.06
5	Chatelle, Camille	7	Owen, Adrian M	0.05
6	Gavaret, Martine	6	Chatelle, Camille	0.03
7	Pruvost-robieux, Estelle	6	Gavaret, Martine	0.03
8	Owen, Adrian M	5	Hermann, Bertrand	0.02
9	Luaute, Jacques	4	Rohaut, Benjamin	0.01
10	Bodien, Yelena G	4	De lucia, Marzia	0.01

**Table 5:** Top 10 co-authors by publication output and centrality.

Rank	Sort by no. of articles published	Citation	Sort by centrality	Centrality
1	Giacino, Joseph T	251	Sitt, Jacobo D	1.13
2	Schnakers, Caroline	162	Faugeras Frédéric	0.92
3	Laureys, Steven	142	Schnakers, Caroline	0.74
4	Schiff, Nicholas D	93	Schiff, Nicholas D	0.7
5	Monti, Martin M	84	Fischer Catherine G	0.65
6	Owen Adrian M	77	Tzovara, Athina	0.42
7	Kondziella, D	73	Bruno Michael A	0.37
8	Bruno, Michael A	72	Naccache L	0.37
9	Teasdale, Graham	71	Gosseries Olivia	0.36
10	Seel, Ronald T	66	Owen Adrian M	0.35

### 3.6 Cited reference analysis

CiteSpace enables visualization of citation counts through co-occurrence analysis, effectively identifying high-impact papers and their knowledge connections within a field. Using “References” as nodes with a Top *N* = 20 threshold and Web Explorer’s network pruning function, the resulting keyword co-occurrence network comprises 219 nodes, 352 connections, and a network density of 0.0147 (Figure 4C). Table 6 lists the top 10 most cited papers (21–30), with the European Academy of Neurology guidelines on the diagnosis of coma and other disorders of consciousness being the most frequently cited, authored by Kondziella D et al. in 2020 (21). These guidelines present the latest evidence on the diagnosis of DOC, summarizing data from bedside examination techniques, functional neuroimaging, and electroencephalography (EEG). This work is followed by a review titled “Recovery from Disorders of Consciousness: Mechanisms, Prognosis, and Emerging Therapies,” published by Edlow BL in 2021 (22). This review focuses on the mechanisms of recovery from impaired consciousness (DOC) during the acute and subacute-to-chronic stages and discusses recent advances in detecting and predicting consciousness recovery. This work also describes developments in pharmacological and electrophysiological therapies that are creating new opportunities to improve the lives of patients with DOC. The 10 articles (Table 6) address the definition, diagnosis, prognosis, treatment mechanisms, multimodal (behavioral, EEG, and fMRI) assessment, nursing recommendations, central regulatory interventions, efficacy of sensory stimulation, fMRI-based functional connectivity of local brain networks, and accuracy of different EEG indicators in evaluating consciousness status, serving as the knowledge foundation in this research field. Among these, sensory stimulation falls within the scope of occupational therapy, encompassing primarily visual, tactile, gustatory, and olfactory stimuli. The cited references are published predominantly between 2014 and 2020, reflecting the enduring influence of classical literature in this field. This may also be attributed to the time lag required for citing recent research accumulation and the current scarcity of high-quality evidence-based studies. Therefore, future research efforts should focus on exploring new research hotspots and conducting more high-level studies to advance the field. Notably, the top 10 most central papers all exhibit a centrality ≥0.1, indicating their pivotal “bridging” role in knowledge dissemination (Table 6) and their pivotal position in the network (21, 26, 28, 29, 31–36). Particularly noteworthy are the European Academy of Neurology guidelines on the diagnosis of coma and other disorders of consciousness, which simultaneously demonstrate high-level citation frequency and centrality (21). This finding reflects its role not only as a foundational knowledge base in the field but also as a critical nexus connecting diverse research directions. By integrating diagnostic criteria, multimodal assessment methods, and other research themes, these guidelines play a pivotal role in promoting knowledge flow and convergence within the field.

**Table 6:** Top 10 cited references by citation frequency and centrality.

Rank	Sort by no. of articles published	Citation	Sort by centrality	Centrality
1	Kondziella D, 2020, EUR J NEUROL, V27, P741, DOI 10.1111/ene.14151	53	Wannez S, 2017, ANN NEUROL, V81, P883, DOI 10.1002/ana.24962	0.42
2	Edlow BL, 2021, NAT REV NEUROL, V17, P135, DOI 10.1038/s41582-020-00428-x	35	Demertzi A, 2015, BRAIN, V138, P2619, DOI 10.1093/brain/awv169	0.4
3	Giacino JT, 2018, NEUROLOGY, V91, P450, DOI 10.1212/WNL.0000000000005926	35	Naccache L, 2015, BRAIN, V138, PE395, DOI 10.1093/brain/awv190	0.35
4	Thibaut A, 2019, LANCET NEUROL, V18, P600, DOI 10.1016/S1474-4422(19)30031-6	28	Kondziella D, 2020, EUR J NEUROL, V27, P741, DOI 10.1111/ene.14151	0.34
5	Claassen J, 2019, NEW ENGL J MED, V380, P2497, DOI 10.1056/NEJMoa1812757	26	Cossy N, 2014, FRONT PSYCHOL, V5, P0, DOI 10.3389/fpsyg.2014.00155	0.33
6	Wannez S, 2017, ANN NEUROL, V81, P883, DOI 10.1002/ana.24962	25	Morlet D, 2014, BRAIN TOPOGR, V27, P467, DOI 10.1007/s10548-013-0335-5	0.32
7	Giacino JT, 2014, NAT REV NEUROL, V10, P99, DOI 10.1038/nrneurol.2013.279	19	Cheng LJ, 2018, FRONT NEUROL, V9, P0, DOI 10.3389/fneur.2018.00826	0.31
8	Cheng LJ, 2018, FRONT NEUROL, V9, P0, DOI 10.3389/fneur.2018.00826	15	Risetti M, 2013, FRONT HUM NEUROSCI, V7, P0, DOI 10.3389/fnhum.2013.00775	0.29
9	Demertzi A, 2015, BRAIN, V138, P2619, DOI 10.1093/brain/awv169	14	Faugeras F, 2011, NEUROLOGY, V77, P264, DOI 10.1212/WNL.0b013e3182217ee8	0.28
10	Engemann DA, 2018, BRAIN, V141, P3179, DOI 10.1093/brain/awy251	14	Wannez S, 2018, NEUROPSYCHOL REHABIL, V28, P1350, DOI 10.1080/09602011.2017.1310656	0.28

Citation literature keyword clustering is an extension of citation literature topic keywords and is based on the co-occurrence map of cited literature, where the most representative terms are extracted from the titles and abstracts of cited literature using the LLR algorithm. The generated clustering map (Figure 4D) shows 15 distinct clusters.

Clustering results are typically evaluated using the cluster modularity index (Q value) and the cluster silhouette index (S value). A Q value = 0.8381 (>0.3) indicates that the network clustering structure is significant, with well-defined boundaries for each cluster’s research themes. The S value = 0.9384 (>0.7), which confirms excellent homogeneity within clusters and clear distinctions between clusters, demonstrating reliable clustering results. The primary keywords included in each cluster are shown in Table 7. The clusters cover specific and diverse themes. The largest cluster, #0 Connect-me study, includes primarily intensive care unit (ICU), residual consciousness, late auditory potential, and focal brain, reflecting a focus on critical care scenarios, localized brain injury conditions, and residual consciousness assessment and recovery. The second-largest cluster, #1, encompasses mainly vagus nerve, magnetic modulation, conscious state patients, p300 correlates, and TDCS responses. This reveals a research trend in which peripheral-central nervous system assessment and modulation techniques are integrated with neuroelectrophysiological evaluation.

**Table 7:** The reference cluster label lists.

Cluster-ID	Cluster name	Size	Top terms (log-likelihood ratio, *p*-level)
#0	Connect-me study	24	Connect-me study (42.6, 1.0E-4); intensive care unit (42.6, 1.0E-4); residual consciousness (42.6, 1.0E-4); late auditory potential (39.02, 1.0E-4); focal brain (39.02, 1.0E-4)
#1	Vagus nerve	18	Vagus nerve (38.03, 1.0E-4); magnetic modulation (38.03, 1.0E-4); conscious state patient (33.24, 1.0E-4); p300 correlate (33.24, 1.0E-4); tdcs response (33.24, 1.0E-4)
#2	Odd-ball paradigm	18	Odd-ball paradigm (43.36, 1.0E-4); behavioral scale (43.36, 1.0E-4); event-related potential (43.36, 1.0E-4); consciousness correlate (39.62, 1.0E-4); coma recovery scale (39.62, 1.0E-4)
#3	Hypothermic postanoxic coma	18	Hypothermic postanoxic coma (47.85, 1.0E-4); eeg responses (40.31, 1.0E-4); alerting sound (40.31, 1.0E-4); cognitive outcome (32.87, 1.0E-4); mismatch negativity (25.54, 1.0E-4)
#4	Brain injury	18	Brain injury (62.76, 1.0E-4); standard eeg (46.9, 1.0E-4); diagnostic process (46.9, 1.0E-4); visual fixation consciousness (41.64, 1.0E-4); differential diagnosis (41.64, 1.0E-4)
#5	Visual pursuit	17	Visual pursuit (48.58, 1.0E-4); conscious state (39.96, 1.0E-4); visual function (38.01, 1.0E-4); diagnostic sign (38.01, 1.0E-4); integrative review (38.01, 1.0E-4)
#6	Study protocol	16	Study protocol (41.57, 1.0E-4); prolonged reduced consciousness (40.17, 1.0E-4); undergoing adjunct tavns therapy (40.17, 1.0E-4); monitoring consciousness (40.17, 1.0E-4); following hemorrhagic stroke (40.17, 1.0E-4)
#7	Randomized clinical trial	14	Randomized clinical trial (54.35, 1.0E-4); randomized controlled clinical trial (48.24, 1.0E-4); traumatic comatose patient (48.24, 1.0E-4); combined effect (48.24, 1.0E-4); sole reflexology massage (48.24, 1.0E-4)
#8	Disclosing result	13	Disclosing result (33.93, 1.0E-4); ethical translation (33.93, 1.0E-4); covert consciousness (25.84, 1.0E-4); auditory steady state response (22.96, 1.0E-4); test–retest reliability (22.96, 1.0E-4)
#9	Pediatric hypoxic–ischemic coma prognostication	10	Pdiatric hypoxic–ischemic coma prognostication (30.24, 1.0E-4); eeg reactivity evaluation practice (30.24, 1.0E-4); north america (30.24, 1.0E-4); eeg reactivity (20.01, 1.0E-4); large inter-rater variability (9.93, 0.005)
#10	Intrinsic functional connectivity	10	Intrinsic functional connectivity (39.93, 1.0E-4); pediatric population (33.58, 1.0E-4); music therapy assessment tool (33.58, 1.0E-4); unresponsive patient (32.32, 1.0E-4); traumatic vegetative state (27.32, 1.0E-4)
#11	Cognitive-motor dissociation	9	Cognitive-motor dissociation (29.9, 1.0E-4); peri-personal space (29.9, 1.0E-4); cognition recovery (26.24, 1.0E-4); cognitive motor dissociation (26.24, 1.0E-4); prospective crossover study (26.24, 1.0E-4)
#12	Observational study	8	Observational study (44.74, 1.0E-4); pediatric sample (44.74, 1.0E-4); influencing recovery (44.74, 1.0E-4); severe tbi (37.19, 1.0E-4); influencing functional outcome (37.19, 1.0E-4)
#13	Auditory addball paradigm	6	Auditory oddball paradigm (34.59, 1.0E-4); bedside detection (34.59, 1.0E-4); investigating patient (26.61, 1.0E-4); information processing (25.54, 1.0E-4); unresponsive patient (24.07, 1.0E-4)
#14	Unconscious cognitive processing	6	Unconscious cognitive processing (21.78, 1.0E-4); event-related eeg potential (21.78, 1.0E-4); proof (10.78, 0.005); using eeg (10.78, 0.005); disorder (10.78, 0.005)

### 3.7 Keyword analysis

**Figure 5:**
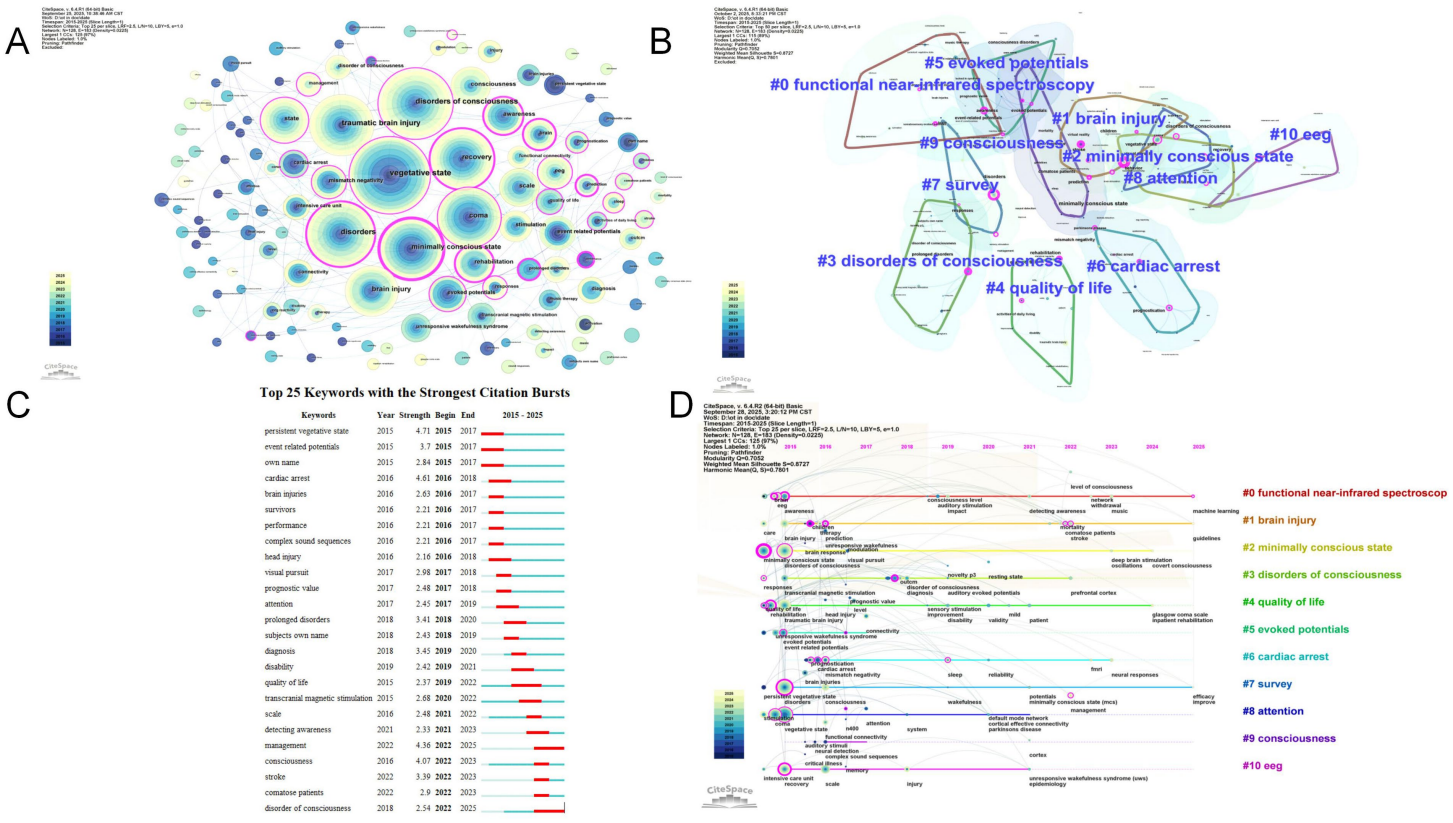
Bibliometric keyword analysis in research. **(A)** High-frequency keyword network. **(B)** Keyword cluster mapping. **(C)** Visualization of the top 25 keywords with the strongest citation bursts. **(D)** Keyword timeline analysis.

CiteSpace was employed to conduct keyword co-occurrence analysis, which visualizes the frequency of different keywords and reveals core themes and hot topics in the research field. When “keywords” were used as nodes, the threshold was set at Top *N* = 25, and the network trimming function was applied. The resulting keyword co-occurrence network graph comprises 128 nodes, 183 connections, and a network density of 0.0225 (Figure 5A). Hot topics in a research field are represented by high-frequency keywords, while the position and significance of related research content within the field are indicated by high-centrality keywords. The top 20 most frequently occurring keywords, as shown in Table 8, are vegetative state, disorders of consciousness, disorders, traumatic brain injury, recovery, coma, minimally conscious state, brain injury, state, consciousness, scale, rehabilitation, awareness, event-related potentials, evoked potentials, stimulation, mismatch negativity, EEG, brain, and unresponsive wakefulness syndrome. Among these, the keywords with the highest-level centrality are minimally conscious state, awareness, rehabilitation, and prediction. The 20 keywords with the highest intermediary centrality all have a centrality ≥0.1 (Table 8), indicating their pivotal role in the network.

Keyword clustering extends keyword co-occurrence mapping by extracting representative terms from cited articles using the LLR algorithm. This method categorizes and names highly correlated keyword groups to generate a visualized clustering map, effectively revealing the knowledge structure and research hotspot distribution across different thematic sectors within a research field. As shown in Figure 5B, the clustering comprises 11 distinct groups. These clusters systematically delineate the comprehensive knowledge framework of occupational therapy in the field of consciousness disorders, encompassing multiple dimensions such as assessment methods, types of consciousness disorders, complications, rehabilitation goals, and research paradigms.

Clustering results are typically evaluated using the cluster modularity index (Q value) and the cluster silhouette index (S value). The Q value of 0.7052 (>0.3) indicated a significant network clustering structure with well-defined boundaries for each cluster’s research themes. The S value of 0.8727 (>0.7) confirmed excellent homogeneity within clusters and clear distinctions between clusters, demonstrating reliable clustering results. The primary keywords in each cluster are listed in Table 9. The clusters included specific yet diverse themes. The largest cluster, #0 functional near-infrared spectroscopy (fNIRS), included primarily functional near-infrared spectroscopy, minimally conscious state, event-related potentials (ERPs), functional magnetic resonance imaging (fMRI), and visual stimuli, clearly pointing to the cutting-edge direction of using fNIRS, EEG, and fMRI multimodal evaluation methods for objective assessment and mechanism exploration of consciousness states. The second-largest cluster, #1, included mainly brain injury, virtual reality, EEG reactivity, minimally conscious state, and interoception, indicating that rehabilitation intervention technologies for patients with consciousness disorders such as brain injury are integrated with emerging technologies such as virtual reality and fundamental neuroscience concepts such as intrinsic perception, heralding innovation and deepening in rehabilitation models.

**Table 8:** Top 20 keywords by frequency and centrality.

Rank	Sort by no. of articles published	Frequency	Sort by centrality	Centrality
1	Vegetative state	136	Minimally conscious state	0.54
2	Disorders of consciousness	122	Awareness	0.36
3	Disorders	111	Rehabilitation	0.35
4	Traumatic brain injury	92	Prediction	0.3
5	Recovery	86	Prolonged disorders	0.23
6	Coma	86	Disorders	0.22
7	Minimally conscious state	85	Performance	0.22
8	Brain injury	66	Brain	0.21
9	State	46	Recovery	0.2
10	Consciousness	37	Evoked potentials	0.2
11	Scale	36	Cardiac arrest	0.19
12	Rehabilitation	35	Mismatch negativity	0.16
13	Awareness	34	Quality of life	0.16
14	Event related potentials	30	Responses	0.16
15	Evoked potentials	29	Sroke	0.16
16	Stimulation	28	Vegetative state	0.15
17	Mismatch negativity	26	Coma	0.14
18	EEG	25	Event-related potentials	0.14
19	Brain	21	Disorders of consciousness	0.13
20	Unresponsive wakefulness syndrome	21	State	0.13

**Table 9:** The keyword cluster label lists.

Cluster-ID	Cluster name	Size	Top terms (log-likelihood ratio,p-level)
#0	Functional near-infrared spectroscpoy	14	Functional near-infrared spectroscopy (fnirs) (10.46, 0.005); minimally conscious state (10.09, 0.005); erp (6.8, 0.01); fmri (6.8, 0.01); visual (5.22, 0.05)
#1	Brain injury	14	Brain injury (13.32, 0.001); virtual reality (10.92, 0.001); eeg reactivity (5.49, 0.05); minimally conscious state (5.44, 0.05); |interoception (5.07, 0.05)
#2	Minimally conscious state	13	Minimally conscious state (36.8, 1.0E-4); disorders of consciousness (31.34, 1.0E-4); behavioral assessment (7.56, 0.01); visual pursuit (6.71, 0.01); covert consciousness (5.28, 0.05)
#3	Disorders of consciousness	13	Disorders of consciousness (10.74, 0.005); prefrontal cortex (9.93, 0.005); disorder of consciousness (9.05, 0.005); minimally consciousness state (6.28, 0.05); auditory evoked potentials (6.04, 0.05)
#4	Quality of life	13	Quality of life (40.68, 1.0E-4); activities of daily living (25.77, 1.0E-4); traumatic brain injury (22.22, 1.0E-4); intervention (17.99, 1.0E-4); outcome (9.09, 0.005)
#5	Evoked potentials	11	Eevoked potentials (17.02, 1.0E-4); 40 hz (10.38, 0.005); event-related potentials (7.73, 0.01); prognosis (7.27, 0.01); auditory system (6.71, 0.01)
#6	Cardiac arrest	11	Cardiac arrest (28.52, 1.0E-4); mismatch negativity (13.75, 0.001); therapeutic hypothermia (13.72, 0.001); targeted temperature management (13.72, 0.001); coma (8.72, 0.005)
#7	Survey	10	Survey (8.76, 0.005); critical care (6.9, 0.01); persistent vegetative state (5.55, 0.05); brain injuries (5.51, 0.05); tbi (5.18, 0.05)
#8	Attention	10	Attention (6.49, 0.05); traumatic brain injury (4.9, 0.05); doc (4.63, 0.05); vegetative state (4.13, 0.05); cardiac arrest (4.08, 0.05)
#9	Consciousness	9	consciousness (9.09, 0.005); p600 (8.98, 0.005); mmn (8.98, 0.005); semantics (8.98, 0.005); erps (8.98, 0.005)
#10	EEG	7	EEG(6.11, 0.05); approximate entropy (4.92, 0.05); affect (4.92, 0.05); matrix (4.92, 0.05); skilled nursing facilities (4.92, 0.05)

The term “burst” denotes a sudden surge in frequency over a defined period. Burst word analysis helps identify keywords that exhibit high frequency fluctuation rates and rapid growth patterns, enabling further exploration of frontier research areas and disciplinary trends (37). The top 20 keywords with the most pronounced citation bursts from 2015 to 2025 are displayed in Figure 5C. The blue line delineates the time intervals, while the red line marks the keyword outbreak events, indicating these keywords as pivotal themes during the specified period. Among these terms, “persistent vegetative state” (strength 4.71) and “cardiac arrest” (strength 4.61) were the strongest emergent terms in the early stages, reflecting that research at the time may have focused primarily on specific etiologies (e.g., cardiac arrest) and typical states of consciousness (e.g., vegetative state). Moreover, the emergence of keywords such as “event-related potentials” (strength 3.7), “visual pursuit” (strength 2.98), and “own name” (strength 2.84) indicated that researchers were actively employing neurophysiological and behavioral assessment methods, including event-related potentials, visual tracking, and responses to one’s own name, to provide a basis for the objective evaluation and treatment of patients with impaired consciousness. In 2018 and 2019, the prominence of “prolonged disorders” (strength 3.41) and “diagnosis” (strength 3.45) marked the increasing emphasis on chronic consciousness disorders and diagnosis. Concurrently, “quality of life” (strength 2.37) and “disability” (strength 2.42) emerged as new hotspots, indicating that research began to focus more on core rehabilitation objectives such as functional impairments and quality of life in patients. The emerging terms in recent years indicate the cutting-edge directions in this field. The two persistent terms, “management” (strength 4.36) and “disorder of consciousness” (strength 2.54), underscore that systematic management of patients with consciousness disorders has become a current research trend.

The timeline visualization in CiteSpace further illustrates the clustering along the timeline (Figure 5D). The timeline graph, with time on the horizontal axis, intuitively presents the temporal evolution of research topics in the field of consciousness disorders from 2015 to 2025 and further highlights keywords that exhibit a sharp increase in citation frequency or occurrence frequency within specific time periods.

The research timeline of #0 functional near-infrared spectroscopy (fNIRS) extends from 2015 to 2025. The early stage (2015–2020) focused on fundamental applications for consciousness level detection associated with nodes such as “consciousness level” and “awareness detection.” The later stage (2021–2025) shifted toward technology integration, linked to keywords such as “machine learning,” aiming to achieve intelligent and precise detection of consciousness states. The timeline of #1 brain injury spans from 2015 to 2025. In the early stage (2015–2020), theoretical research on brain injury was concentrated, with nodes centered on “brain injury” and “prediction,” and mechanisms of injury occurrence and prevention were analyzed. The later stage (2021–2025) transitioned to prognosis and clinical guidelines, featuring keywords such as “guidelines” and “stroke,” reflecting a shift from “mechanistic research” to “clinical intervention guidelines.” Current research trends emphasize the deep integration of multimodal neurotechnology and artificial intelligence, progressing from mechanistic research and prevention to clinical rehabilitation management, with interdisciplinary convergence becoming increasingly evident.

## 4 Discussion

### 4.1 Research status

This bibliometric analysis explored the research progress related to occupational therapy for impaired consciousness over the past decade using CiteSpace and Bibliometrix. In recent years, particularly since 2019, there has been a significant upward trend in the number of publications, peaking in 2023. This surge is likely closely associated with the COVID-19 pandemic. COVID-19 affects the nervous system, respiratory system, skeletal muscles, and nearly all bodily systems, leading to impaired consciousness, motor dysfunction, cognitive impairment, and reduced ability to perform activities of daily living. A retrospective study of 90 severe/critical COVID-19 patients in Sichuan Province revealed that approximately 20% of patients experienced impaired consciousness (38). Additionally, this impaired consciousness prolongs ICU stay, during which patients often face increased risks of mechanical ventilation, sedative use, and nosocomial infections (e.g., multidrug-resistant pulmonary infections), exacerbating cerebral hypoxia and inflammatory responses, thereby further inhibiting consciousness recovery (39). Therefore, we speculate that the global COVID-19 pandemic may have led to a surge in the number of patients with impaired consciousness, highlighting the necessity and safety of early rehabilitation and occupational therapy for impaired consciousness, thereby accelerating the rapid expansion of clinical practice and research in this field.

The field of occupational therapy for consciousness disorders has currently formed a research landscape centered in the United States with Europe as a collaborative hub. The United States dominates the academic network with the highest publication volume and an intermediary centrality of 0.87. Institutions such as the French National Institute of Health and Medical Research (INSERM) and the University of Liège in Belgium, although not at the top in terms of publication volume, have become key bridges for transatlantic collaboration. European countries (Italy, France, the UK, and Belgium) have formed secondary clusters through close cooperation, while China, despite demonstrating lasting influence on citation half-life, still falls short in international collaboration contributions. Top scholars in this field are concentrated in Europe and America (e.g., Belgium and the United States), with core journals dominated by the United States and the UK. We speculate tha this pattern may be attributed to strong policy support and substantial financial investments in healthcare in these countries, such as the United States BRAIN Initiative, the EU’s “Human Brain Project,” and special funding from the National Institutes of Health, which may have facilitated enhanced collaboration through clinical neuroscience alliances at institutions such as Harvard Medical School and INSERM (40). However, current exchanges and collaborations remain limited; for example, in terms of geographical participation from Asian and South American countries, in disciplinary areas, there is insufficient integration between basic neural mechanisms and clinical rehabilitation, and at the level of research scholars, a core group of authors has yet to form. Future research would benefit from interdisciplinary integration, such as developing multimodal neuroimaging–electrophysiological assessment tools to improve the accuracy of diagnosis and prognosis prediction, designing precision occupational therapy based on neuroplasticity, and leveraging the sustained advantages of China’s clinical resources and citations to construct a global multicenter trial network, promoting cross-domain collaboration in the closed loop of “brain mechanism exploration–rehabilitation technology translation–clinical validation.”

### 4.2 Research hotpots and trends

#### 4.2.1 Multimodal assessment techniques

Advances in multimodal assessment techniques, including neuroimaging and electrophysiological technologies, have demonstrated significant promise in improving the clinical evaluation of DOC. Extensive comprehensive studies have explored changes in brain activity patterns in DOC patients by examining both stimulus-induced and spontaneous brain activity, providing valuable insights for clinical diagnosis and prognosis. Nevertheless, reaching consensus on neuroimaging biomarkers for DOC patients remains a challenge (41). Current task paradigms include active, passive, and resting-state approaches, primarily utilizing task-based fMRI (42), resting-state fMRI (43), electroencephalography (EEG) (44, 45), positron emission tomography (PET), functional ultrasound imaging (46), task-based fNIRS (47), and resting-state fNIRS (48) for assessment and prognostic evaluation. Functional near-infrared spectroscopy (fNIRS) has shown great potential in the clinical evaluation of DOC owing to its dual advantages of portability and high spatial resolution. Existing studies have systematically investigated the effects of acupuncture, motor imagery, and auditory stimulation on cortical activation in DOC patients using fNIRS technology (49, 50). Researchers have noted that this technique can more accurately capture changes in consciousness, thereby facilitating more precise diagnostic and therapeutic decisions and providing patients with more efficient and accurate rehabilitation services. However, the application of fNIRS in assessing the severity of DOC and prognostic evaluation requires further investigation. What’s more, with the advancement of artificial intelligence, machine learning has been widely applied in this field, enhancing the accuracy of assessments. For instance, machine learning is utilized to integrate multisource neural data features, replacing manual efforts to achieve waveform and network recognition of key indicators, thereby simplifying complex assessments and prognostic judgments through mapping and classifiers (29, 51–53). These evaluation techniques and algorithms provide a tool for bedside stratified evaluation.

Furthermore, although there are diverse approaches to evaluating consciousness disorders, current research on the efficacy of occupational therapy for consciousness disorders relies primarily on scale-based assessments. These assessments focus mainly on consciousness levels, somatic dysfunction, mental health, cognitive dysfunction, pain, activities of daily living (ADL) and quality of life, as well as disability severity, cognitive function, disease severity, DOC duration, and mechanical ventilation duration. With the advancement of multimodal neuroelectrophysiological and neuroimaging evaluation techniques for consciousness disorders, their application in evidence-based research on occupational therapy should be promoted, which would facilitate the exploration of the neural mechanisms underlying occupational therapy and enable the design of precision-oriented occupational therapy interventions, thereby providing a scientific basis for implementing occupational therapy protocols.

#### 4.2.2 Multisensory stimulation

Multisensory stimulation is a crucial component in the field of occupational therapy for impaired consciousness and is aimed at improving patients’ level of consciousness. Such stimulation specifically includes auditory stimulation, visual stimulation, tactile stimulation, olfactory stimulation, and pain stimulation. Multisensory stimulation has a positive effect on the level of consciousness, pain intensity, and agitation in patients with impaired consciousness and can serve as an effective strategy in medical centers (54–58). However, clinical evidence regarding their therapeutic effects remains inconsistent (59, 60). Although previous studies have conducted meta-analyses on the efficacy of sensory stimulation and family-centered sensory stimulation therapy for patients with DOC, they suffer from limitations such as insufficient timeliness, lack of refined classification of sensory stimulation types, and failure to examine the effects of different intervention types and dosages on rehabilitation outcomes (56, 61). A longstanding theoretical perspective suggests that regulated sensory stimulation may facilitate positive therapeutic outcomes; however, patients exposed to undifferentiated bombardment of sensory information lose information processing ability owing to habituation (62). Rehabilitation therapists, who are proficient in sensory modulation principles, can provide controlled sensory stimuli calibrated to higher thresholds of reticular neurons, thereby increasing cortical activity (63). Therefore, high-quality randomized controlled trials targeting the parameters of recent intervention protocols should be conducted in the future to provide evidence-based references for the development of more effective multisensory stimulation approaches for patients with consciousness disorders.

#### 4.2.3 Intelligent rehabilitation devices in occupational therapy

With the advancement of technology, researchers have begun to focus on the application of intelligent rehabilitation devices in occupational therapy for impaired consciousness (64). Virtual reality (VR) is an immersive environmental technology that simulates multisensory interactions through computers, providing comprehensive stimuli such as visual, auditory, and tactile inputs. Its application in the treatment of impaired consciousness (DOC) has demonstrated significant potential. Through multisensory integration and neural modulation mechanisms, VR can effectively activate residual cognitive functions, assess, and improve patients’ level of consciousness. Studies have shown that multisensory stimulation based on the Neurowave system can significantly enhance motor and cognitive functions in patients with microconsciousness states (MCSs), with effects superior to those of traditional sensory stimulation (SS) (65). Through dynamic environmental videos, immersive VR induces autonomic nervous responses, leading to a significant increase in electrodermal activity (EDA) in DOC patients, indicating that sympathetic nerve activation is associated with consciousness recovery (66, 67). In the rehabilitation of children with impaired consciousness, VR combined with conventional therapy can temporarily improve Glasgow Coma Scale (GCS) and Coma Recovery Scale (CRS-R) scores, but long-term improvements in social functioning (GOS-E Peds) require further validation (68). Future research should expand the sample size, integrate multimodal neuroelectrophysiological and imaging techniques to explore the mechanisms of VR, and develop personalized intervention plans. The long-term efficacy and standardized application of VR still require further investigation.

#### 4.2.4 Brain-computer interface in occupational therapy

Brain-Computer Interface (BCI) technology is a novel type of rehabilitation therapy that is controlled by neural activity and capable of real-time feedback. This technology establishes a communication and control channel between the brain and the external environment without relying on peripheral nerve and muscle conduction by decoding individual psychological intentions and converting neural activity into internal or external command signals to achieve direct interaction between the brain and the external environment (69). Currently, BCI rehabilitation systems have been widely applied in the rehabilitation training of patients with neurological disorders. However, in the field of consciousness disorders, the application of BCI is focused primarily on auxiliary diagnosis and prognosis evaluation (70, 71). Future exploration is warranted for its application in occupational therapy rehabilitation training for patients with consciousness disorders.

Robot-assisted gait training promotes changes in neuroplasticity, recovery of somatic function, and improvement of consciousness in patients with impaired consciousness through repetitive training and feedback while demonstrating a certain level of safety (72). However, future research should address ethical challenges while resolving issues related to safety and feasibility.

#### 4.2.5 Animal-assisted training

Animal-assisted training (AAT) refers to a method that introduces animals (such as dogs and horses) to assist in rehabilitation therapy, thereby enhancing patient participation and treatment efficacy. AAT can significantly improve the engagement and emotional-behavioral positivity of patients with impaired consciousness while reducing fatigue and the frequency of training interruptions (73, 74). In the future, AAT research should further refine intervention protocols, including the species of animals, training modalities, and application scenarios, to more precisely accommodate the needs of different patients. Additionally, exploring the long-term rehabilitation effects of AAT on patients with impaired consciousness and its synergistic role in other therapeutic strategies, such as multisensory stimulation, is essential to provide more personalized and effective rehabilitation plans for these patients. This approach would further enhance treatment outcomes and improve patients’ quality of life.

#### 4.2.6 Focusing on the emotional and psychological well-being of family members

In addition to focusing on the emotional and psychological well-being of family members, researchers have begun to address the emotional and social challenges posed by cognitive impairment to patients’ families and have proposed the need for family-oriented care approaches that provide understanding, empathy, holistic support, and collaboration (75). However, relevant intervention studies have not yet been conducted.

#### 4.2.7 Personalized occupational therapy

Finally, on the basis of the results of this analysis, we found that the level of consciousness significantly influenced the acceptance and efficacy of occupational therapy. Patients with lower levels of consciousness may require earlier sedation adjustments to facilitate early occupational therapy interventions, whereas those with higher levels of consciousness necessitate more standardized sedation management to avoid compromising early rehabilitation. Additionally, patients with longer hospital stays, older age, more comorbidities, and higher levels of consciousness are more likely to accept occupational therapy implementation (76). This finding suggests that occupational therapists and related personnel should identify the most likely beneficiaries, optimize resource allocation, and ensure timely provision of necessary occupational therapy services. Future research on occupational therapy should further explore specific intervention strategies for patients with varying levels of consciousness to achieve more personalized and efficient rehabilitation outcomes.

### 4.3 The strength of evidence supporting OT interventions in DOC

From a research design perspective, most current literature mainly relies on retrospective studies and observational studies, with a shortage of high-quality randomized controlled trials. Some studies lack scientific randomization, blinding, and control designs, which leads to higher bias risks and limited evidence reliability.

Regarding sample size, clinical recruitment of DOC patients is inherently difficult. Most studies use small sample sizes and single-center data, potentially resulting in insufficient statistical power and limited generalizability of findings to the broader DOC population.

In terms of OT intervention quality, some protocols lack standardized procedures, fail to fully embody the OT-centered practice orientation, and show inadequate targeted designs for patient functional participation and daily activity engagement. Significant variations in intervention intensity, frequency, and duration undermine intervention consistency and comparability.

Regarding outcome measures, existing studies predominantly focus on consciousness levels and motor functions. There are limited long-term multidimensional evaluations of OT core dimensions, including occupational participation, role fulfillment, and social functioning, thus failing to comprehensively reflect the true value of occupational therapy. Additionally, certain intervention protocols have poor experimental feasibility in real-world clinical settings due to high equipment requirements, complex operational procedures, and limited patient compliance, leading to weak generalizability.

Despite the aforementioned limitations, existing evidence still partially supports the rationality of applying OT in the treatment of DOC. Multiple studies have demonstrated that OT interventions centered on functional occupational activities, life task training, and participatory rehabilitation can promote perceptual arousal, improve cognitive and motor functions, enhance the potential self-care abilities and social participation potential of DOC patients, and provide sustained environmental stimulation and behavioral reinforcement for consciousness recovery and functional reconstruction. These interventions have clear clinical theoretical foundations and practical evidence. Although the overall strength of evidence remains limited, they have established a certain degree of positive support.

### 4.4 Gaps between technological innovation and practical OT implementation

In recent years, technological innovations in OT for DOC have emerged continuously, such as VR-based cognitive simulation training and intelligent awareness assessment systems, providing new directions for improving treatment precision and patient engagement. However, there remains a significant gap between these technological innovations and real-world OT practices in clinical settings, with most institutions still predominantly relying on traditional occupational therapy approaches. Firstly, the implementation of new technologies faces high barriers, including elevated hardware costs and complex operational requirements. Primary care and general rehabilitation institutions often lack the necessary equipment and technical infrastructure to sustain routine implementation. In contrast, traditional OT focuses on manual activities, life skill training, and basic cognitive tasks—methods that are simple, cost-effective, and better aligned with current clinical realities, thus maintaining its dominant position. Secondly, OT practitioners exhibit insufficient acceptance and mastery of new technologies. Some therapists have developed entrenched traditional clinical mindsets and lack systematic training in advanced techniques, making it difficult to integrate intelligent and digital tools into treatment processes centered on occupational participation. Even when equipped with such devices, technical advantages are often underutilized, ultimately reverting to conventional intervention models. Thirdly, certain technological innovations fail to align with the actual needs of DOC patients. Excessive emphasis on technological sophistication overlooks patients’ consciousness levels, cooperation capabilities, and functional characteristics, resulting in designs that do not closely align with core OT objectives such as occupational practice, functional participation, and activity engagement. This lack of clinical relevance hinders the replacement of traditional occupational therapy in promoting patient autonomy, further limiting the dissemination and application of new technologies.

### 4.5 Strengths, limitations, and future directions

This study provides a comprehensive bibliometric overview of occupational therapy for consciousness disorders, systematically mapping emerging research trends, shifts in global research priorities, and key thematic areas, thereby establishing a foundational framework to advance academic discourse in this field. However, three limitations warrant consideration. First, although we conducted searches in both Chinese and English databases, no relevant studies were found in the Chinese database. Second, the study consisted primarily of articles and reviews, potentially leading to omissions of certain critical themes; and third, the quality of research on OT for DOC requires further improvement; the fourth, there exists a certain gap between technological innovation and actual OT practice. Future research could expand coverage by integrating multiple databases and better capture global trends in occupational therapy for consciousness disorders. Furthermore, future researchers should enhance the quality of their studies to provide higher-quality evidence-based support for OT in patients with DOC. Additionally, the application and promotion of innovative techniques in OT for patients with DOC in clinical practice need further improvement.

## 5 Conclusion

The COVID-19 pandemic has triggered a surge in the number of patients with consciousness disorders, driving the number of studies to peak in 2023 over the past five years. Global research has been dominated by Europe and the United States, with the latter leading the academic network through the highest publication volume and high centrality in intermediate centers, while the former has formed secondary clusters through cross-institutional collaboration. Although China has the advantage of citation durability, international cooperation remains weak. Current hotspots focus on multimodal assessments integrating fNIRS, fMRI, and artificial intelligence, but research on occupational therapy efficacy still relies on traditional scales. Occupational therapy interventions are diverse and include multisensory stimulation, animal-assisted training, intelligent rehabilitation technologies, humanistic care, and personalized occupational therapy pathways. Future efforts should address limitations such as single-database dependence and insufficient interdisciplinary integration, strengthen cross-disciplinary collaboration, explore precision occupational therapy rehabilitation paradigms driven by neural mechanisms for DOC, and enhance the quality of related research as well as promote the application and dissemination of technological innovations in clinical practice.

## Data Availability

The original contributions presented in the study are included in the article/Supplementary material, further inquiries can be directed to the corresponding author.
